# Higher Tablet Use Is Associated With Better Sustained Attention Performance but Poorer Sleep Quality in School-Aged Children

**DOI:** 10.3389/fpsyg.2021.742468

**Published:** 2022-01-03

**Authors:** Karen Chiu, Frances C. Lewis, Reeva Ashton, Kim M. Cornish, Katherine A. Johnson

**Affiliations:** ^1^Melbourne School of Psychological Sciences, University of Melbourne, Parkville, VIC, Australia; ^2^Monash School of Psychological Sciences, Monash University, Clayton, VIC, Australia; ^3^Turner Institute for Brain and Mental Health, Monash University, Clayton, VIC, Australia

**Keywords:** sustained attention, sleep, electronic device use, child development, screen time

## Abstract

There are growing concerns that increased screen device usage may have a detrimental impact on classroom behaviour and attentional focus. The consequences of screen use on child cognitive functioning have been relatively under-studied, and results remain largely inconsistent. Screen usage may displace the time usually spent asleep. The aim of this study was to examine associations between screen use, behavioural inattention and sustained attention control, and the potential modifying role of sleep. The relations between screen use, behavioural inattention, sustained attention and sleep were investigated in 162 6- to 8-year-old children, using parent-reported daily screen use, the SWAN ADHD behaviour rating scale, The sustained attention to response task and the children’s sleep habits questionnaire. Tablet use was associated with better sustained attention performance but was not associated with classroom behavioural inattention. Shorter sleep duration was associated with poorer behavioural inattention and sustained attention. Sleep quality and duration did not act as mediators between screen usage and behavioural inattention nor sustained attention control. These findings suggest that careful management of the amount of time spent on electronic screen devices could have a beneficial cognitive impact on young children. The results also highlight the critical role of sleep in enhancing both behavioural attention and sustained attention, which are essential for supporting cognitive development and learning.

## Introduction

With exposure to digital media increasing over the last decade (Mullan, 2018) and newer devices offering interactive features, novelty, and repeated positive reinforcement (Huang et al., 2020), there are growing concerns about the developmental consequences of screen time on the emerging cognitive landscape in children as they start school for the first time (Ghandour et al., 2021). Of particular concern is the impact of screen time, time spent unrelated to learning activities that may affect attentive behaviour in the classroom and a child’s ability to focus, avoid distraction and learn. Attention is most often measured behaviourally with parent, teacher and self-reported questionnaires in which the child is compared with other children of the same age. An example is from the Strengths and Weaknesses of ADHD symptoms and normal behaviour (SWAN) in which the child is rated on how well they “Remain focused on task (does not stare into space or daydream)” (Swanson et al., 2012). One problem with relying on behavioural manifestations of inattention is that the external state may not necessarily reflect accurately the corresponding internal state of attention. A child looking at the teacher may in fact be attending to inner thoughts about last night’s football game. In recent years, a flurry of studies has begun to investigate the role of cognitive attention in facilitating classroom learning (Trautmann and Zepf, 2012; Shannon et al., 2020). The pioneering work of Posner and colleagues has been instrumental in identifying a set of separable but interconnected neural systems (Posner and Petersen, 1990; Petersen and Posner, 2012) responsible for responding quickly and accurately to incoming information by selecting relevant and ignoring irrelevant stimuli (Chica et al., 2014), and the sustained attention system that helps regulate attention control over time (Mahone and Schneider, 2012). The sustained attention system is the focus of this current research. To date, there are very few studies that have examined the interplay between behavioural inattention and sustained attention in young children and the impact of screen time. Of the few published studies on behavioural inattention, there is evidence to suggest that greater screen time – time spent viewing content on television, tablet, phone, and videogames, is associated with behavioural inattention in children (Swing et al., 2010; Gentile et al., 2012; McNeill et al., 2019; Tamana et al., 2019; Thoma et al., 2020). The few published studies on the association between screen use and sustained attention have all measured response inhibition performance using Go/No-Go tasks, reflecting the capacity to withhold responses to rare No-Go stimuli while maintaining attentional focus to appropriately respond to infrequently occurring Go stimuli (Hwang et al., 2019). A study by Carson et al. (2017) found no association between total daily screen time and target detection on the Fish-Shark Go/No-Go task in children aged between 2.5 and 5 years. Similarly, McNeill et al. (2021) found that daily screen use time was not associated with response inhibition accuracy on the Fish-Shark Go/No-Go task in children aged 3 to 5 years old. In contrast, 3–5-year-old children who spent higher amounts of time (>30 min) using applications on portable devices (e.g., tablet, laptops, mobiles and handheld game devices) displayed poorer response inhibition performance, measured using a Go/No-Go task from the Early Years Toolbox one year later in a longitudinal design, than children who spent lower amounts of time (<30 min) using applications (McNeill et al., 2019). Notably, the total electronic media use and programme viewing were not associated with any measures of executive functioning or prosocial behaviours at follow-up (McNeill et al., 2019). Further clarification is required to understand whether screen use is associated with sustained attention performance in primary school-aged children, and whether there are differing effects across different screen devices.

Staying alert and focused in class are critical skills for successful early learning (Steele et al., 2012; Alavi et al., 2019). In addition to screen use, growing evidence highlights the critical role of sleep as a fundamental mechanism facilitating behavioural attention and sustained attention in the classroom (Paavonen et al., 2009; Gruber et al., 2012; Davidson et al., 2021). Sleep is essential for brain development and functioning in children (Jan et al., 2010; Gruber et al., 2012). Poor sleep is associated with less efficient attentional processing (Hoyniak et al., 2015) and poorer sustained attention in children (Davidson et al., 2021). Tasks relying on sustained attention are highly sensitive to sleep deprivation (Doran et al., 2001). Following sleep deprivation, wake-state instability occurs where sleep-initiating mechanisms interfere with the effort to stay awake, leading to increasingly variable cognitive performance (Rogers et al., 2003). Wake-state instability is reflected through a general slowing of response times, increased omission and commission errors and an increased time-on-task effect in cognitive attention tasks (Lim and Dinges, 2008). Experimentally manipulated sleep restriction is associated with longer mean and increased lapses in response time, increased response time variability and commission errors (Kuula et al., 2015) and increased omission errors (Gruber et al., 2011; Davidson et al., 2021) on vigilance and continuous performance tasks in children.

Excessive screen use is associated with poorer sleep in children, including shorter sleep duration, poor sleep quality and excessive daytime sleepiness (Carter et al., 2016). One explanation is offered by the displacement hypothesis (Cain and Gradisar, 2010), which suggests that screen use displaces time that otherwise would have been spent sleeping (Hale and Guan, 2015). Research, however, has largely focused on television use and sleep, whereby longer parent-reported television viewing has been consistently associated with shorter sleep duration and poorer sleep quality as measured by both parent reports (Cespedes et al., 2014; Marinelli et al., 2014) and actigraphy devices in children (Helm and Spencer, 2019). The use of newer devices, including computers, videogames, tablets and mobile phones, is associated with even greater adverse sleep outcomes in later childhood and adolescence (Hale and Guan, 2015), although less research has focused on younger school-aged children. The extent to which screen time and sleep difficulties impact both cognitive and behavioural attention control is not clearly understood. One possibility is that diminished sleep may be one of the mediating mechanisms underlying the associations between increased screen usage and poor attentional control (Barlett et al., 2012; Guerrero et al., 2019). No studies however have explored the mediating effect of sleep on sustained attention.

Given that screen technology is continually updating and devices have become an everyday tool for children even in the preschool years, there is an imperative to identify the associations between screen use, behavioural inattention, sustained attention and the potential modifying role of sleep. The first hypothesis was that longer screen use (i.e., television, tablet, smartphone and videogame console use independently) would predict increased (1a) inattentive behaviours, (1b) poorer sustained attention and (1c) poorer sleep in children. The second hypothesis was that poorer sleep would be associated with both behavioural inattention and sustained attention difficulties. The third hypothesis was that sleep quality and duration would act as mediators between screen use and both behavioural inattention and sustained attention control.

## Materials and Methods

### Participants

This study was part of a larger three-year longitudinal study assessing attention and academic outcomes in children (Johnson et al., 2020a). This study used data from the second year of the study. Participants were 234 children aged 6.5–8.3 years old. Exclusion criteria were (1) parents not completing the sleep survey – 63 children, (2) parents not completing the screen use survey – an additional 1 child, (3) making 60 or more omission errors on the Sustained Attention to Response Task (SART), indicating a failure to complete the task appropriately – 5 additional children, (4) refusing to complete the SART – 1 additional child and (5) a standard score on the Raven’s Coloured Progressive Matrices (RCPM) of less than 70 – no child met this exclusion. Two children were unavailable for testing of the RCPM and were excluded. In total, 72 children were excluded. The final sample (*n* = 162) is described in Table 1. Nineteen children were reported by their parents as having at least one clinical diagnosis. Nine children were reported as having a diagnosis of anxiety, six with Autism Spectrum Disorder, two with hearing loss, two with Expressive Language Disorder, two with Attention Deficit Hyperactivity Disorder and one with depression. These children were included as this is a normative sample from mainstream schooling.

This study was conducted with the approval of the University of Melbourne Human Research Ethics Committee, the Department of Education and Training (DET) and all Victorian dioceses of the Catholic Education Office (CEO). Principals, classroom teachers and parents/caregivers provided written consent, and children provided verbal assent.

### Materials and Procedure

Parents or guardians completed a survey containing demographic information, screen use and Children’s Sleep Health Questionnaire Abbreviated. Teachers completed the Strengths and Weaknesses of ADHD Symptoms and Normal Behaviour (Swanson et al., 2012). Children were tested individually in a quiet room during school hours at the end of their second year (October–December 2018) or the start of their third year at school (February–June 2019). Children completed four half-hour sessions of activities, one-on-one with one of several trained researchers, as part of the larger project. Children completed one half-hour session a day; a small number of children completed two sessions a day, with a break between sessions, due to logistics. The order in which children completed the sessions, and the activities within the sessions, was counterbalanced.

#### Screen Use

For each device (television, tablet, smartphone and videogame console), parents reported ‘how long does your child use these devices on a typical school day’ on a 6-point scale (never, a few times a week but not daily, up to one hour a day, 1–2 h a day, 2–3 h a day and 3+ hours a day).

#### Children’s Sleep Habits Questionnaire Abbreviated

The CSHQ-A (Owens et al., 2000; NICHD SECCYD, 2016) consisted of 22 items regarding sleep difficulties in children (e.g., ‘child is afraid of sleeping in the dark’, ‘child wakes up more than once during the night’, ‘child snores loudly’ and ‘child is restless and moves a lot during sleep’). Parents reported their child’s typical sleep patterns in the past week on a 5-point scale ranging from ‘always’ (occurred every night in the past week) to ‘never’. Parents also recorded their child’s usual sleep duration, combining both night time sleep and naps.

#### Strengths and Weaknesses of ADHD Symptoms and Normal

The SWAN (Swanson et al., 2012) is an 18-item scale capturing both attention skills and problems in children. Teachers answered on a 7-point scale ranging from ‘far below average’ (−3) to ‘far above average’ (+3), where 0 represented ‘average’ relative to other children the same age. There were two subscales: inattention and hyperactivity-impulsivity. Teachers did not complete the SWAN for 19 children.

#### Ravens Coloured Progressive Matrices Test

The Ravens Coloured Progressive Matrices Test (RCPM; Raven et al., 1990) was used to measure non-verbal intelligence in the first year of assessments to exclude children with estimates of non-verbal general cognitive ability of <70.

#### Sustained Attention to Response Task

**Figure 1:**
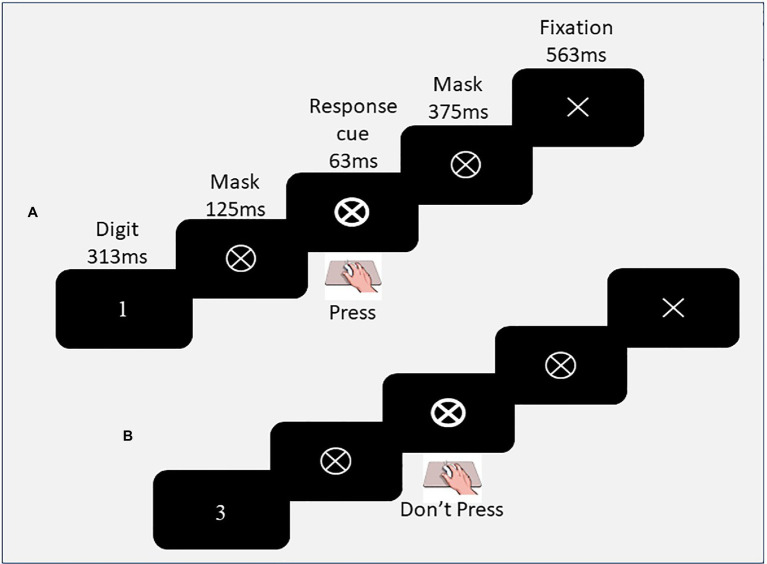
The sustained attention to response task sequence of events and timings. Figure depicts **(A)** a go trial, requiring a response to the presentation of the go-digit 1, and **(B)** a no-go trial, requiring the withholding of a response to the no-go digit 3. In this Fixed version of the task, the digits 1–9 are presented within a fixed sequence, 25 times. Participants were asked to respond on the response cue.

The Fixed version of the SART (Bellgrove et al., 2005) was presented on a 15-inch laptop using E-Prime software. Each trial consisted of a single digit (1–9) presented for 313 ms, followed by a mask (crossed circle) for 125 ms, a response cue (bolded crossed circle) for 65 ms, a second mask for 375 ms and a fixation cross for 563 ms (see Figure 1), with an onset-to-onset interval of 1,439 ms. Digits were presented in the centre of the laptop screen in five randomly selected font sizes (48, 72, 94, 100 and 120 point; heights 12-29 mm) in a repeating fixed ascending order. Children were asked to press the mouse when the circle/cross flashed after presentation of each digit, except for the no-go digit (3). Prior to commencing the task, participants were asked to re-explain the task to ensure they understood the instructions. There were 36 practice trials (with 4 No-Go trials), and 225 experimental trials (25 No-Go trials). The experimental task duration was 5.5 min.

### Data Analysis

#### Screen Use

Responses from the screen use items were recoded into numerical values, where ‘never’ was converted to 0, ‘a few times a week but not daily’ was converted to 0.25, ‘up to one hour a day’ to 0.5, ‘1–2 h a day’ to 1.5, ‘2–3 h a day’ to 2.5 and ‘3+ hours a day’ to 3.

#### Children’s Sleep Habits Questionnaire Abbreviated

Sleep quality was estimated by summing the responses, with a higher score reflecting worse sleep quality. For omitted sleep duration responses but completed weekday/weeknight sleep and wake times, an average duration was used. Sleep quality and duration were correlated, *r* = −0.27 [CI: −0.41, −0.12], *t*(158) = −3.56.

#### Symptoms and Normal Behaviour

Attention and hyperactivity-impulsivity subscale scores were separately summed and divided by the number of items (9). The SWAN Inattentive and Hyperactive–Impulsive measures were highly correlated, *r* = 0.81 [CI: 0.75, 0.86], *t*(141) = 16.5; therefore, the SWAN Inattention measure alone was used for subsequent analyses as the behaviours rated on this subscale were most relevant to the research question.

#### Sustained Attention to Response Task

The method of Johnson et al. (2007) was followed for both initial data preparation and the FFT and ex-Gaussian analyses (Johnson et al., 2020b). Sustained attention was assessed using measures of accuracy (e.g., omission and commission errors) and speed of responses (e.g., mean response time and standard deviation of response time) in the SART. Counts of commission and omission errors were calculated per participant. Commission errors (number of times a child responded to a no-go stimulus) are thought to reflect symptoms of hyperactivity or impulsivity, while omission errors (number of times a child failed to respond to a go stimulus) represent symptoms of inattention (Barkley, 1991; Halperin et al., 1991). Mean response time (MRT) and standard deviation of response time (SDRT) were also calculated. MRT represents information processing speed (Luce, 1986), while SDRT assesses response style consistency, where larger variability reflects more lapses in attention (Robertson et al., 1997). MRT and SDRT, however, do not provide details on changes in performance at certain timescales or over the course of the SART (Lewis et al., 2017). Thus, this present study also computed a Fast Fourier Transform (FFT) spectra of RT data and ex-Gaussian analysis to provide richer indices of response variability, than MRT and SDRT alone (Johnson et al., 2007).

#### FFT Data Preparation

RTs less than 100 ms (Luce, 1986) and RT to the No-Go target 3 were linearly interpolated, using the RTs immediately preceding and following that trial. Individual RT data were detrended by subtracting any linear component of the time series.

#### FFT Spectra Computation

Using MATLAB, RT data were analysed using Welch’s averaged, modified periodogram method. The full time series was divided into seven sections of 75 data points each, with an overlap of 50. Any section containing more than 5 omission errors in total was excluded; the omission errors did not have to be consecutive. Any participant with more than three out of the seven segments missing was excluded – 14 children were excluded. Each segment was Hamming-windowed and zero-padded to length 450 before computing the FFT, and the spectra derived were averaged across the seven segments. The MATLAB script used is available at the Open Science Framework at https://osf.io/ntwy7/. Using the method of Johnson et al. (2007), the FFT spectrum was divided into frequencies above and below 0.0772 Hz, which is the reciprocal of the ISI (1.439 s) by 9 digits in one SART cycle. This division at 0.0772 Hz marks the fast frequency area under the spectra (FFAUS) and slow frequency area under the spectra (SFAUS). FFAUS reflects variability within one cycle of digits 1 to 9, reflecting momentary fluctuations in RT, while SFAUS reflects variability greater than one cycle, reflecting slow gradual change in RT over the task. Therefore, FFAUS likely reflects momentary fluctuations in attention, while SFAUS reflects a gradual decline in attention due to declining arousal over the task.

As RT may be positively skewed due to occasional very long responses, an ex-Gaussian model was used to extend upon a normally distributed model to capture these very slow responses (Luce, 1986). The maximum-likelihood-based distribution-fitting routines of (Lacouture and Cousineau, 2008) were used to fit mu, sigma and tau to each participant’s data set using Matlab. Mu and sigma represent the centrality and spread of the Gaussian distribution, respectively, while tau is the exponential component and represents the tail in the skewed distribution. When fitted to RT data, it denotes very long response times (Johnson et al., 2015) and is thought to reflect attentional lapses (Tarantino et al., 2013).

#### Statistical Analyses

Each measure was calculated per participant. The data were analysed using R version 3.6.3 (R Core Team, 2012), R Studio version 1.2.5042, the *lme4* package (Bates et al., 2020) and the *mediation* package (Tingley et al., 2014). An analysis of the sample by sex and a comparison of the included and excluded participants was conducted using *t-*tests and chi-square. Generalised linear mixed-effects models with a Poisson distribution were used to analyse the SART count data (errors of commission and omission) with the beta coefficients exponentiated. Linear mixed-effects models were used to analyse the remaining data. School was included as a random effect to minimise the effect of children within the same school being more like each other than children between schools.

**Figure 2:**
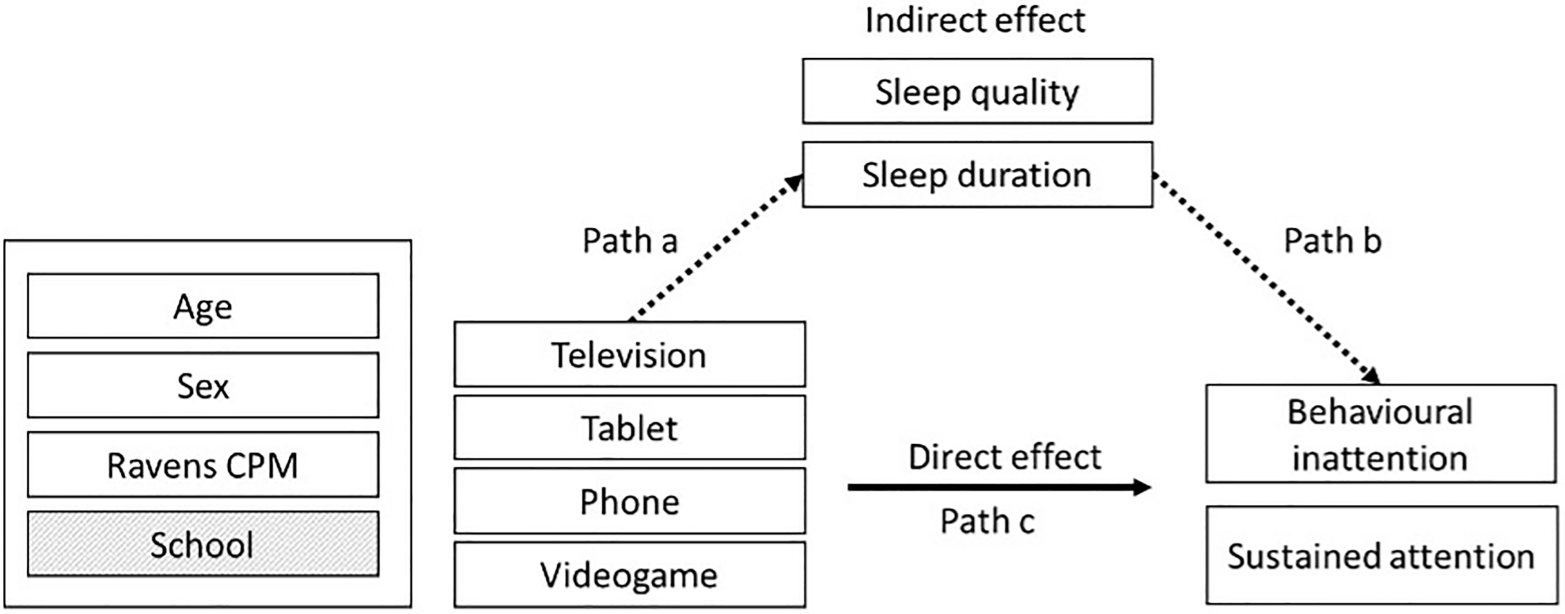
The paths analysed in the statistical models.

Age, Sex and RCPM (as fixed effects) and School (random effect) were included in the base model given past research has indicated strong associations with attention and sleep outcomes (Gordon et al., 1990; Biggs et al., 2013; Lewis et al., 2017; Tremolada et al., 2019). To test hypothesis one, each screen type measure (television, tablet, phone and videogame) separately was added to the base model as a fixed effect to explain variance in the SWAN and SART measures (path c), and sleep quality and duration (path a; see Figure 2). To test hypothesis two, sleep quality and duration, separately, were added as fixed effects to the base model to explain variance in the SWAN and SART measures (path b). To test hypothesis three, if any of the screen time measures explained a significant amount of variance in attention (path c) and sleep quality and/or sleep duration (path a), further analyses were used to test whether sleep quality and duration explained a significant amount of variance in the attention measures (path b) while controlling for the screen time measures.

Predictors were considered significant if the confidence interval (CI) of the beta coefficient did not contain the value of zero for most outcome measures, and one for the commission and omission errors. Visual inspection of residual plots did not reveal any obvious deviations from homoscedasticity. Corrections for multiple testing were not performed due to the exploratory nature of this research.

## Results

### Descriptive Statistics

**Figure 3:**
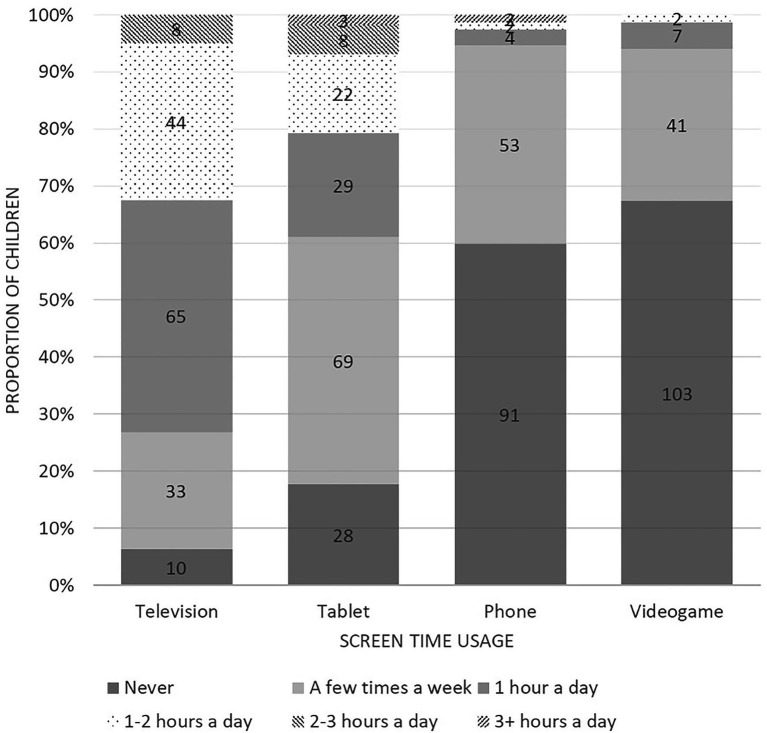
The proportion of children who spent time on each device during weekdays graphed by device type.

The descriptive statistics, SART performance, SWAN ratings, sleep scores and device use are described in Table 1. There was no significant difference in age between the boys and girls, *t*(136) = −1.89, CI [−0.26, 0.01]. The proportion of children who spent time using each device is graphed by device type in Figure 3. Television (94%) and tablet (82%) were most popular with the children. Less than half the participants used a phone (40%) or videogame device (33%).

**Table 1:** The descriptive statistics for the sample and each of the outcome measures.

Measure	All, mean (SD), count of data, [range]	Boys, mean (SD), count of data, [range]	Girls, mean (SD), count of data, [range]
Number	162	96	66
Age (years)	7.3 (0.4), 162, [6.5, 8.3]	7.4 (0.4), 96, [6.5, 8.3]	7.3 (0.4), 66, [6.5, 8.1]
Commissions	7.1 (4.1), 162, [0, 21]	7.7 (4.5), 96, [0, 21]	6.3 (3.3), 66, [0, 15]
Omissions	13.6 (10.0), 162, [0, 56]	13.0 (10.6), 96 [1, 56]	14.4 (9.1), 66, [0, 37]
Mu	441 (172), 160, [116, 912]	423 (163), 94, [116, 815]	467 (181), 66, [214, 912]
Sigma	146 (68), 160, [14, 326]	139 (68), 94, [14, 326]	155 (68), 66, [38, 296]
Tau	145 (94), 160, [6, 359]	146 (93), 94, [4, 374]	144 (96), 66, [6, 359]
FFAUS	559 (256), 160, [68, 1,282]	556 (274), 94 [91, 1,282]	563 (230), 66, [68, 1,258]
SFAUS	2,384 (1364), 160, [275, 7,047]	2,232 (1366), 94, [275, 6,840]	2,601 (1342), 66, [342, 7,047]
SWAN Inattention	−0.4 (1.1), 143, [−3.0, 2.8]	−0.2 (1.2), 80, [−3.0, 2.3]	−0.6 (1.1), 63, [−2.8, 2.8]
Sleep quality	20 (9), 162, [2, 46]	20 (9), 96, [2, 46]	20 (9), 66, [6, 45]
Sleep duration (hrs)	10.3 (1.0), 160, [5, 12.5]	10.2 (1.1), 95, [5, 12.3]	10.5 (0.9), 65, [7, 12.5]
Television time	0.8 (0.6), 160, [0, 2.5]	0.8 (0.7), 95, [0, 2.5]	0.7 (0.6), 65, [0, 2.5]
Tablet time	0.6 (0.7), 159, [0, 3]	0.6 (0.7), 94, [0, 3]	0.6 (0.8), 65, [0, 3]
Phone time	0.2 (0.4), 152, [0, 3]	0.2 (0.4), 91, [0, 3]	0.1 (0.4), 61, [0, 3]
Videogame time	0.1 (0.2), 153, [0, 1.5]	0.1 (0.3), 93, [0, 1.5]	0.1 (0.1), 60, [0, 0.5]

There was no significant difference between the boys and girls in terms of television viewing time *t*(149) = −0.82 [−0.29, 0.12], tablet use, *t*(118) = 0.79 [−0.15, 0.34] or phone use, *t*(128) = −0.31 [−0.15, 0.11]. The boys spent more time playing videogames than the girls *t*(145) = −2.28, [−0.13, −0.01].

Children had an average sleep duration of 10.3 h. Twenty-one children were reported to ‘rarely’ nap (occurs once a week), and 2 reported they ‘sometimes’ nap (occurs 2 to 4 times a week).

T-tests and chi-squares tests between included and excluded children revealed that excluded children were rated more inattentive on the SWAN *t*(90.85) = 3.21 [0.23, 0.98], made more omission errors on the SART *t*(71.97) = 3.37 [6.45, 25.17], and were more likely to have a mental health or neurodevelopmental diagnosis, X2 (1, *N* = 234) = 5.62, *p* = 0.018. There were no differences in age *t*(128.08) = 0.08 [−1.44, 1.55], sex X2 (1, *N* = 234) = 1.29, *p* = 0.999, RCPM score *t*(125.32) = −1.33 [−6.85, 1.34] between included and excluded children. There were also no significant differences in other SART variables, including commission errors *t*(131.20) = −0.52 [−1.46, 0.86], mu *t*(95.30) = −1.66 [−96.43, 8.73], sigma *t*(81.56) = −0.67 [−32.42, 16.03], tau *t*(94.06) = 1.54 [−6.54, 51.40], FFAUS *t*(93.24) = 0.60 [−55.84, 103.90] and SFAUS *t*(80.92) = 1.40 [−146.77, 835.57].

### Hypothesis One

#### Summary of Significant Results

Longer screen time did not predict inattentive behaviour, commission errors, mu, tau, FFAUS or SFAUS. More time spent using tablets and playing videogames was associated with less omission errors made. More time using tablets was associated with smaller sigma and poorer sleep quality.

#### SWAN Inattention

Base model (path C). Age, beta coefficient − 0.26 [CI: −0.71, 0.20] explained little variance in the SWAN Inattention score. Boys (*n* = 80, m − 0.21, SD 1.16) were rated as more inattentive than girls (*n* = 63, m − 0.61, SD 1.11), 0.48 [CI: 0.12, 0.83]. Children with a lower RCPM estimate were rated as more inattentive than those with a higher RCPM score, −0.25 [−0.38, −0.13]. School SD 0.28 was smaller than the Residual SD 1.05. Television viewing time, 0.01 [−0.27, 0.28], tablet, 0.06 [−0.19, 0.30], phone, 0.16 [−0.38, 0.70] and videogame time, 0.22 [−0.60, 1.04] did not explain any additional variance in SWAN Inattention scores.

Commission errors – Base model (path C). Younger children made more commission errors than older children, exponentiated coefficient 0.81 [CI: 0.69, 0.96]. Boys (m 7.7, SD 4.5) made more commission errors than the girls (m 6.3, SD 3.3), 1.29 [CI: 1.14, 1.47]. The RCPM estimate did not explain a significant amount of variation in commission errors made with the exponentiated coefficient CIs crossing 1, 0.96 [0.92, 1.01]. School SD was 0.19. Television viewing time, 1.06 [0.97,1.16], tablet time, 0.94 [0.86, 1.02], phone time, 0.91, [0.76, 1.09] and videogame time, 0.96 [0.72, 1.26] did not explain a significant amount of variance in commission errors.

**Figure 4:**
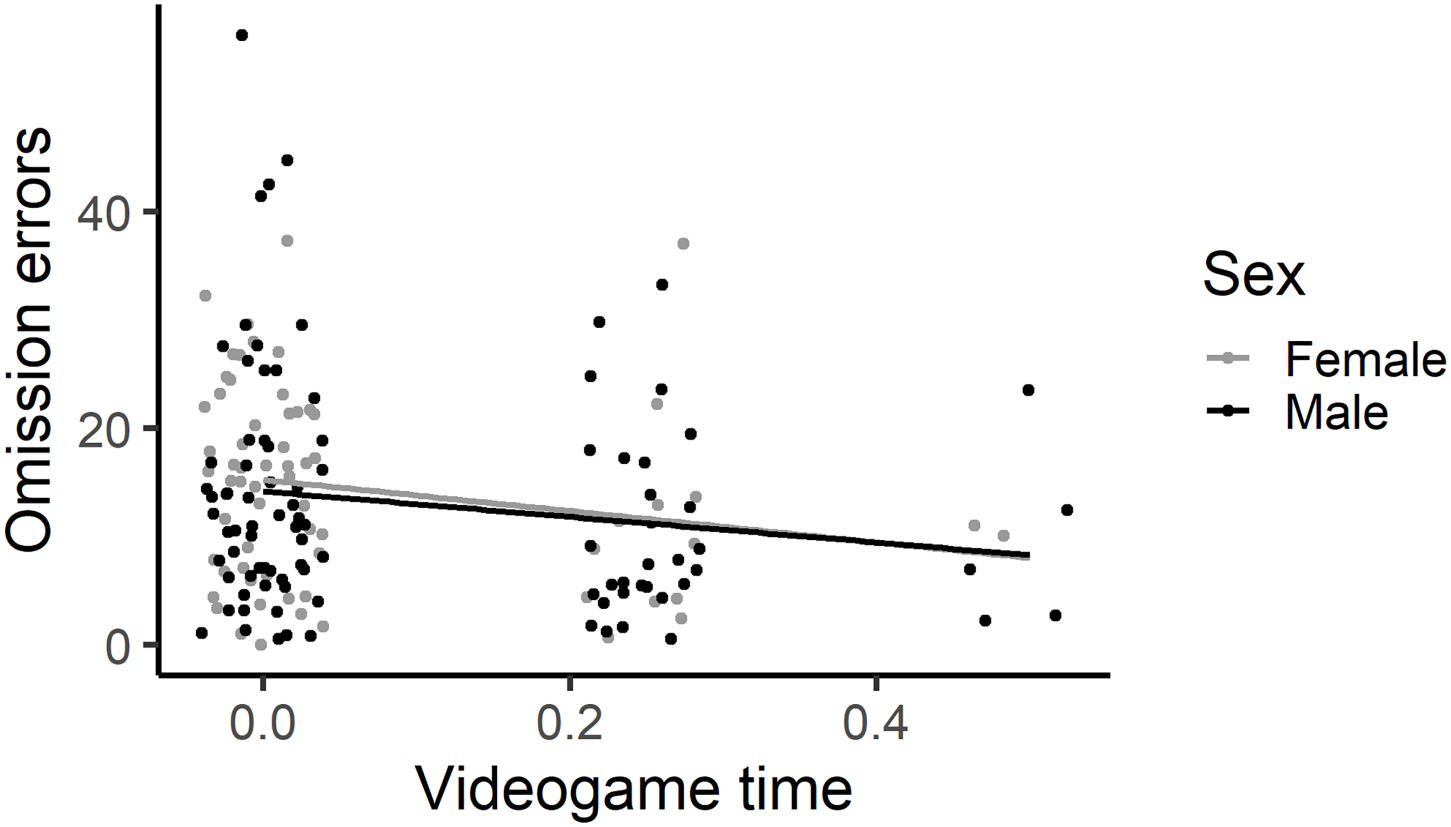
The more time spent playing videogames, the less omission errors made. This graph has each individual’s data point shown, using jittering.

Omission errors – Base model (path C). Younger children made more omission errors than older children, exponentiated coefficient 0.83 [CI: 0.73, 0.95]. Girls (m 14.4, SD 9.1) made more omission errors than the boys (m 13.0, SD 10.6), 0.89 [CI: 0.81, 0.97]. RCPM estimate did not explain a significant amount of variance, 0.97 [0.94, 1.00]. School SD was 0.39. Television viewing time, 0.99 [0.92, 1.06] did not explain a significant amount of variance in omission errors. Children who spent more time using tablets, 0.94 [0.88, 1.00], made less omission errors on the SART. Phone use, 0.93 [0.83, 1.04] was not a significant predictor of omission errors. Children who spent more time playing videogames, 0.58 [0.45, 0.75] made less omission errors on the SART. With the two children with higher videogame time usage excluded (1–2 h a day), there was a stronger effect, 0.28 [0.19, 0.41] (see Figure 4).

Mu – Base model (path C). Age, 17 [CI: −47, 81], Sex, −45 [−100, 10] and RCPM, −5 [−24, 13] did not explain a significant amount of variance in Mu. Television viewing time, −20 [−62, 22], tablet use, −28 [−65, 9], phone use, −30 [−101, 41] and videogame time, −84 [−214, 46] did not significantly explain any variance in Mu.

**Figure 5:**
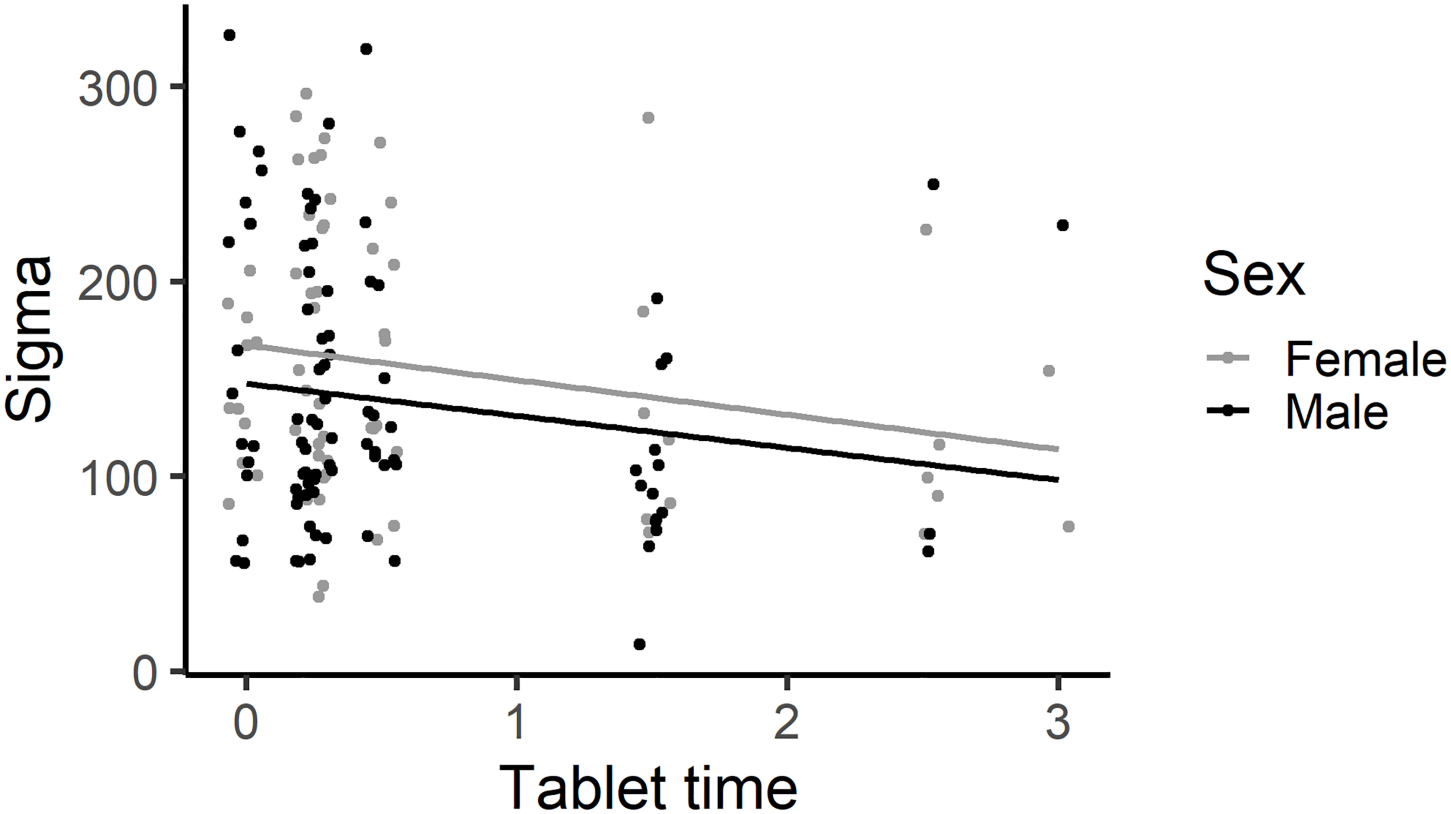
The more time using a Tablet, the less variably the child responded on the SART. This graph has each individual’s data point shown, using jittering.

Sigma – Base model (path C). Age, −4 [CI: −29, 22], Sex, −14 [−36, 8] and RCPM, −5 [−13, 2] did not explain a significant amount of variance in Sigma. Television viewing time, −7 [−24, 9], phone use, −13 [−41, 15] and videogame time, −15 [−66, 36] did not significantly explain any variance in Sigma. The more time spent using a tablet, −18 [−32, −3], the less variably the child responded on the SART (see Figure 5).

Tau – Base model (path C). Age, −20 [CI: −55, 15], Sex, 5 [−25, 35] and RCPM, −2 [−12, 9] did not explain a significant amount of variance in Tau. Television viewing time, 7 [−16, 30], tablet use, 10 [−10, 30], phone use, 9 [−30, 49] and videogame time, 13 [−59, 85] did not significantly explain any variance in Tau.

Fast frequency area under the spectra – Base model (path C). Younger children were more variable in moment-to-moment responding than older children, coefficient − 144 [CI: −237, −50]. Sex, 14 [−66, 94] and the RCPM estimate, −21 [−49, 6] did not explain a significant amount of variance in FFAUS. Television −10 [−71, 51], tablet use −197 [−489, 96], phone use −15 [−119, 89] and videogame time − 100 [−290, 90] did not significantly explain variance in FFAUS.

Slow frequency area under the spectra – Base model (path C). Age, −116 [CI: −623, 392], Sex, −332 [−764, 36] and RCPM, −113 [−262, 36] did not explain a significant amount of variance in SFAUS. Television viewing time, 52 [−279, 384], tablet use −113 [−373, 148], phone use −35 [−605, 535] and videogame time, −369 [−1,415, 676] did not significantly explain any variance in the slow variability measure.

Sleep duration in hours – Base model (path A). Age, 0.03 [CI: −0.39, 0.44], Sex, −0.30 [−0.61, 0.03] and the RCPM estimate, −0.02 [−0.13, 0.09] did not explain a significant amount of variance in sleep duration. Television −0.22 [−0.46, 0.02], tablet −0.14 [−0.36, 0.08] and videogame time − 0.19 [−0.98, 0.60] did not significantly explain variance in sleep duration. The more time spent using a phone, −0.70 [−1.22, −0.18] the shorter the child’s sleep time, but when the two children with high phone usage (3+ hours per day) were excluded, phone use was no longer a significant predictor of sleep duration, −0.29 [1.04, 0.46].

**Figure 6:**
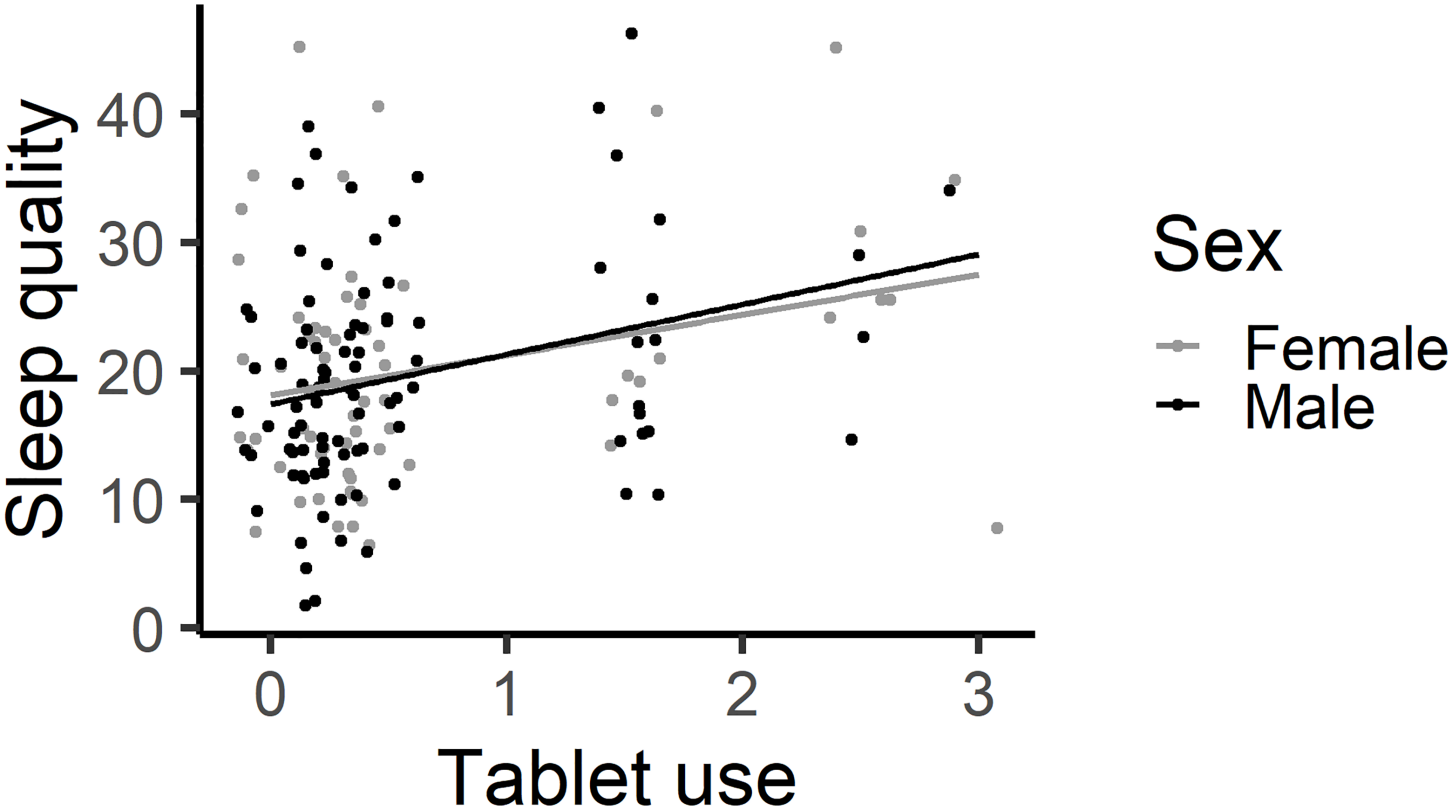
The more time spent on Tablets, the worse one’s sleep quality (higher number represents more sleep issues noted by parents). This graph has each individual’s data point shown, using jittering.

Sleep quality – Base model (path A). Age, −2 [CI: -5, 2], Sex, −0.04 [−2.9, 2.8] and the RCPM estimate, −0.0 [−0.9, 1.0] did not explain a significant amount of variance in sleep quality. Television 0.2 [−1.9, 2.4] and videogame time 6 [−1, 12] did not significantly explain variance in sleep quality. The more time spent on a tablet, 3.5 [1.6, 5.3], the worse the child’s sleep quality (see Figure 6). The more time spent using a phone, 3.9 [0.3, 7.5] the worse the child’s sleep quality, but when the two children with very high phone usage (3+ hours per day) were excluded, phone use was no longer a significant predictor of sleep quality, 5.80 [−0.90, 12.49].

### Hypothesis Two

#### Sleep Duration

**Figure 7:**
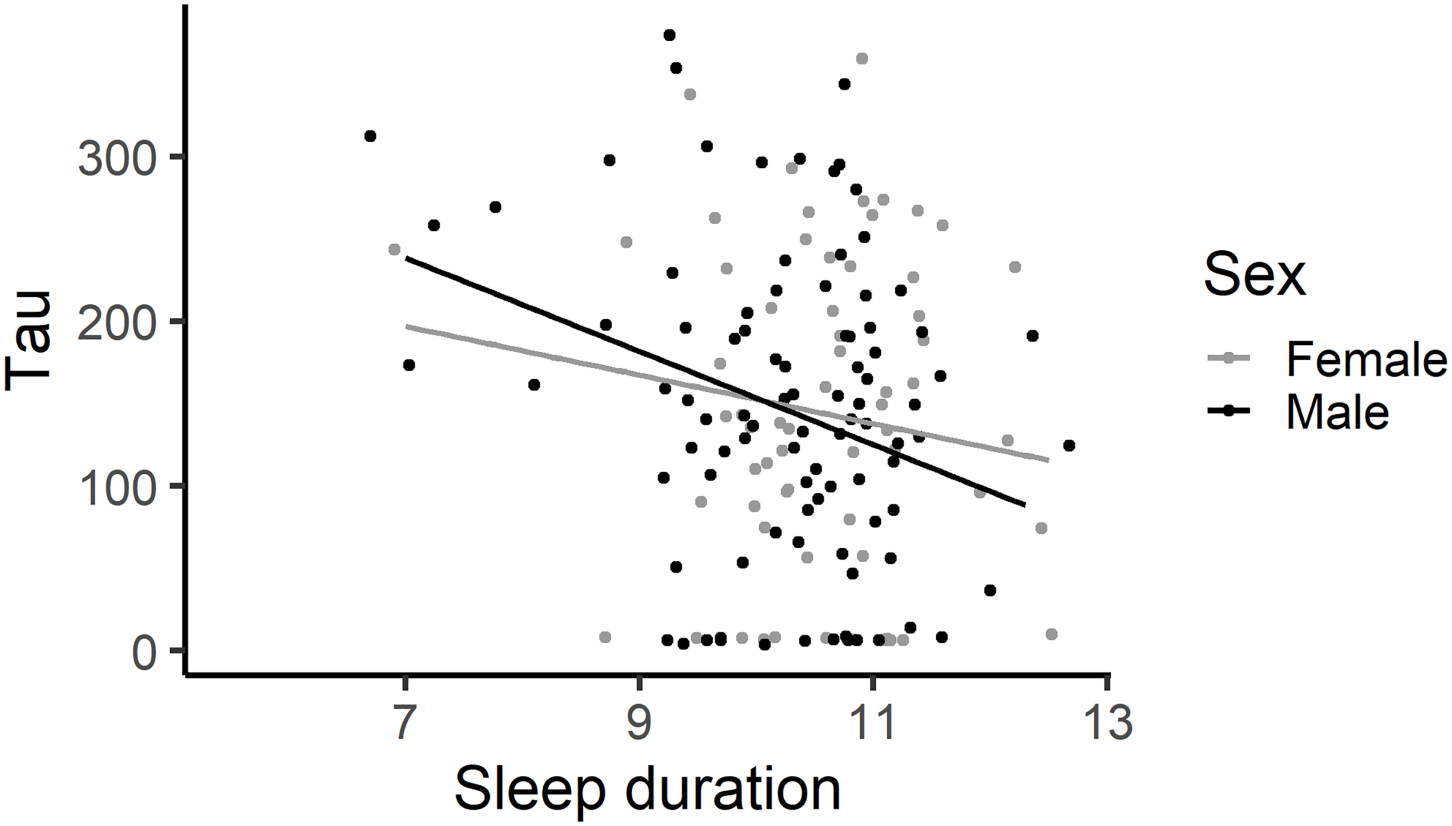
The less time asleep, the more very slow responses (Tau) made on the SART. This graph has each individual’s data point shown, using jittering.

The less time spent asleep, the greater the child’s ADHD inattentive symptoms, −0.21 [−0.41, −0.01], the more omission errors made, exponentiated beta coefficient 0.91 [0.87, 0.95], and the more very slow responses made (tau) on the SART (Figure 7), −24 [−39, −8]. In addition, and in some ways in contrast, the less time spent asleep the faster the child’s response (mu), 43 [15,71], and the less variably the child responded (sigma), 14 [3, 25]. Sleep duration did not explain a significant amount of variation in the number of commission errors made, exponentiated beta coefficient 0.95 [0.92, 1.005], nor in the moment-to-moment variability in responding (FFAUS), −33 [−74, 8] or slow variability in responding (SFAUS), −189 [−412, 34].

#### Sleep Quality

**Figure 8:**
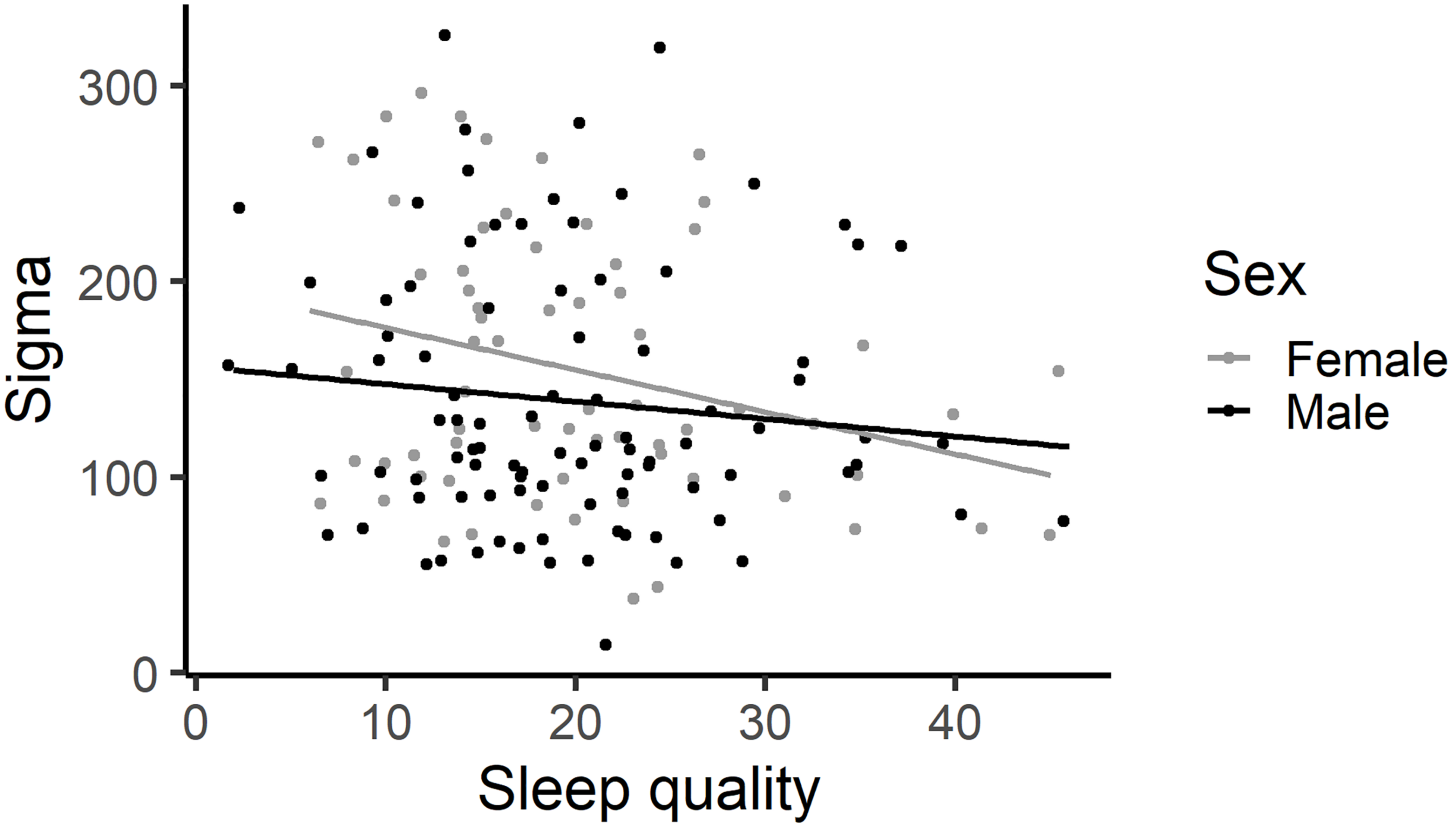
The higher the score on the sleep quality scale, the worse the child’s sleep. The worse a child’s sleep, the less variably the child responded. This graph has each individual’s data point shown, using jittering.

Sleep quality did not explain a significant amount of variance in the ADHD inattentive behaviours, 0.005 [−0.02, 0.03], count of commission errors made, 1.00 [0.99, 1.00], omission errors, 1.00 [0.99, 1.00], mu, −2 [−5, 0.9], tau, 1.54 [−0.11, 3.19], moment-to-moment variability (FFAUS), −0.87 [−5.3, 3.6] or slow variability (SFAUS), −2 [−26, 22]. The worse the child’s sleep quality, the less variably the child responded (sigma), −1.5 [−2.7, −0.3] (Figure 8).

### Hypothesis Three

One mediation model was tested for Hypothesis 3. The effect of time spent using a tablet on the response time variability measure sigma was not mediated *via* sleep quality. The regression coefficient between tablet time and sigma (path c, −17.9 [−32.4, −3.4]), and between tablet time and sleep quality (path a, 3.3 [1.5, 5.2]) were significant. The indirect effect was not significant, (path b, − 1.2 [−2.4, 0.04].

## Discussion

The present study explored the associations between screen use, sleep and attention in children. Longer time spent on screen devices by young primary school children was not associated with increased inattentive behaviours in the classroom, providing little support for hypothesis 1a, and was not associated with poorer sustained attention, providing little support for hypothesis 1b. Indeed, higher levels of tablet and videogame use were associated with better sustained attention in young school children. Specifically, children who spent more time using tablets and videogames made fewer omission errors and those who spent more time using tablets responded less variably on the SART. Higher tablet use was associated with poorer sleep quality, but not sleep duration, providing partial support for hypothesis 1c. Shorter sleep duration was associated with poorer behavioural attention control in the classroom and a more haphazard cognitive attention performance on the SART, supporting hypothesis 2. The haphazard performance involved making more omission errors and occasional very slow responses (tau) but with a faster (mu) and less variable (sigma) response style. Echoing the sleep duration result, the worse a child’s sleep quality, the less variably the child responded (sigma). The association between shorter sleep duration and poorer behavioural and cognitive attention control was independent of the direct effect between increased tablet usage and reduced sigma, providing no support for hypothesis three. Sleep quality and duration do not act as mediators between screen usage and behavioural and cognitive attention control. These results suggest two separate effects – tablet use is associated with better sustained attention on a dull, repetitive task and that decreased sleep duration has a negative effect on classroom behaviour and is associated with a disordered response style on the SART.

### Screen Usage Was Not Associated With Increased Inattentive Behaviours in the Classroom in This Sample

Television, tablet, phone and videogame use were not significant predictors of behavioural inattention in this sample of children. This finding contrasts with previous research that have found associations between inattentiveness and exposure to television of 2 or more hours per day (Ozmert et al., 2002; Miller et al., 2007; Swing et al., 2010), and videogame usage of more than 1 h per day (Swing et al., 2010) in samples of children aged 4–17. Consistent with previous research in children (Yu and Baxter, 2016; Rhodes, 2017), televisions (94%) and tablets (82%) were the most popular devices within our sample of 7 to 8 year olds. In contrast, less than half of the participants used phones (40%) or videogame devices (33%). The proportion of children who use smartphones and videogames typically increases with age due to greater access to these devices (Yu and Baxter, 2016; Rhodes, 2017). For example, Yu and Baxter (2016) found that children aged 6–7 years play videogames for an average of 14 min on weekdays, while 12–13 year olds play an average of 88 min. Similarly, Rhodes (2017) found that only 17% of primary school-aged children used smartphones every day, compared to 71% of adolescents. The Australian 24-Hour Movement Guidelines for Children and Young People (Department of Health, 2019) recommended that children and youth aged 5–17 years limit sedentary recreational media exposure to a maximum of two hours a day. Although the categorical nature of our data limited the ability to assess the children’s overall screen device use, it appeared that majority of children in this study were adhering to these guidelines on individual device use across television (95%), tablet (94%), phone (99%) and videogame devices (100%). Thus, it may be possible that content and time spent across these digital domains are not enough to gauge any association with behavioural inattention and media exposure within our sample.

Boys and girls did not differ in time spent on television, tablet and phone devices. Notably, there was a significant sex difference in videogame use, with boys spending more time playing videogames than girls. Gender differences in game play across childhood have been well established in the literature, suggesting that boys are significantly more likely than girls to play electronic games (Homer et al., 2012; Yu and Baxter, 2016). Notably, girls made more omission errors but fewer commission errors than boys on the SART. Girls may have adopted a more risk-averse approach to completing the SART, where they demonstrate more inhibitory control and cautious responding than boys (Cross et al., 2011).

### Tablet Usage Was Associated With Good Sustained Attention Control in This Sample

In terms of cognitive attention, screen use was not associated with any negative effects on sustained attention performance in this sample of children. Instead, time spent using tablets was associated with good concentration on the deliberately boring SART task – reduced omission errors and lower sigma in these young school children. This may occur through practice and the exercising of the sustained attention system. Tablet usage appears to be transferring to the skill of being able to concentrate on a deliberately boring task. This finding is consistent with a study by Dye and Bavelier (2010) which found that youth aged 7–17 years who played shooter games displayed an enhanced ability to orient and maintain attention across visual attention tasks. Videogames may improve hand–eye coordination and increase visual processing (Dye et al., 2009), which may subsequently improve task performance in children. Our results indicate that tablets may also act to improve sustained attention functioning through practice. Further research is required to understand these underlying mechanisms in tablet use and the exact tasks the children were performing on the tablets. Given that sustained attention has been found to predict academic achievement (Steele et al., 2012), and development of future ADHD symptoms (Martin et al., 2012), our findings suggest that tablets and videogame device use may present as a unique avenue for training specific cognitive functions, such as attention, and potentially improve skills at school and in daily life.

### Tablet Usage Was Associated With Reduced Sleep Quality, but Not Sleep Duration

Higher levels of tablet usage predicted poorer sleep quality in children. This finding is consistent with a study by Dube et al. (2017) which found that tablet use was associated with poorer sleep quality and efficiency in 10-11-year-old children. Screen usage was not associated with sleep duration however. The sleep displacement hypothesis proposes that the more time that is spent on screen devices, the less time there is available for sleep (Hale and Guan, 2015). In this current study, more time spent on tablets was not associated with fewer hours of sleep. This finding provides little support for the displacement hypothesis. Instead, our finding that tablet use was associated with poorer sleep quality may support the content arousal hypothesis (Cain and Gradisar, 2010). Screen activities that are alerting may increase levels of physiological and psychological arousal, leading to difficulties in falling asleep and reduced REM sleep, and ultimately poorer sleep quality (Higuchi et al., 2005). Given the important implications of sleep in brain and cognitive development (Jan et al., 2010; Gruber et al., 2012), greater awareness by parents and health professionals of the detrimental impact of screen use just before bedtime is needed and this adds to the growing importance of the need to remove screen time prior to bedtime for all children of primary age.

### Sleep Duration Is Very Important for Behavioural and Cognitive Attention Control

Sleep duration is an important factor in classroom behavioural attention control. Disrupted or poor sleep often manifests as distractibility, poor concentration, and disruptive behaviours in children (O’Brien, 2009). These behaviours are thought to mimic the core inattentive, hyperactive, and impulsive symptomology of ADHD (Dahl, 1996; Spruyt and Gozal, 2011). At a behavioural level, shorter sleep duration predicted greater teacher-rated symptoms of inattention. This is consistent with previous research that has found that insufficient sleep was associated with higher levels of behavioural attention problems in typically developing children (Gruber et al., 2012); (age range 7–11 years); (Paavonen et al., 2009); and (age range 5–6 years). These results suggest that insufficient sleep may be related to tiredness and daytime difficulties in attention, which highlight the need to promote healthy sleep to optimise school functioning of young children.

Shorter sleep duration was also associated with cognitive attention control characterised by faster and more consistent (less variable) responding but with occasional attention lapses that result in increased omission errors and occasional very slow responses. The sleep quality measure echoed the sigma association with sleep duration, with reduced sleep quality associated with a more consistent response style. The haphazard response style on the SART represents a novel finding and highlights the importance of capturing difference facets of performance during the task. Previous research has identified an association between shorter sleep duration and increased omission errors (Gruber et al., 2011; age range = 7–11 years) and attentional lapses on vigilance tasks (Peters et al., 2009; mean age = 10.6 years). Insufficient sleep is particularly relevant in long, simple and monotonous tasks like the SART that are dependent on maintaining attention to the task. Sleep deprivation is thought to decrease alertness and attention through slowed responses, attentional lapses and wake-state instability (Alhola and Polo-Kantola, 2007). The increase in omission errors (failure to respond to a target stimulus) and very long responses (tau) are thought to reflect attentional lapses and microsleeps influenced by insufficient sleep (Goel et al., 2009). This haphazard response style may reflect an attempt by the child to perform well on the task, and so respond quickly and with consistency, but the occasional attention lapses became apparent through this taxing 5.5 min task. These findings indicate that reduced sleep duration interferes with both cognitive and behavioural attention.

### Sleep Does Not Act as a Mediator Between Screen Use and Attention Control

The third hypothesis was that sleep quality and duration would act as mediators between screen use and both behavioural and cognitive attention control. Tablet use explained a significant amount of variance in sigma on the SART (path c), and in sleep quality (path a). Hence, further analyses were conducted to assess whether sleep quality explained a significant amount of variance in sigma while controlling for the tablet use (paths a and b). The effect of time spent using a tablet on sigma was not mediated by sleep quality. This finding stands apart from relevant but not analogous past studies that found that sleep was a significant mediator between media exposure on televisions, videogames and computers, and behavioural attention problems in typically developing children and adolescents (Barlett et al., 2012; Guerrero et al., 2019). Comparing these studies with the current results, path a and b results were consistent, in that screen use was associated with poorer sleep, while sleep was associated with increased attention problems, although their measures were based on behavioural attention, rather than cognitive attention. In contrast, while their path c indicated that screen use predicted poorer behavioural attention as measured by the Child Behaviour Checklist (Guerrero et al., 2019), the current results found that tablet use, specifically, was associated with less variable sigma performance (or better sustained attention) on the SART. This novel finding indicated that screen use may have differential effects on behavioural and cognitive attention.

### Limitations and Future Research

A limitation of this study was its cross-sectional nature, which restricts the ability to make causal inferences. As interactions between sleep, attention and screen use may develop non-linearly and dynamically throughout childhood, further longitudinal studies are needed to explore these interactions across development. Our study also relied on parental reports of sleep. Future research could use objective measures (e.g., actigraphy and polysomnography) to supplement subjective data to provide a more comprehensive understanding about the impacts of screen use on sleep. Our screen use data were also limited to time spent across independent devices. Future research could include a measure of total screen time, and explore areas, such as content, use before bed, and dual-tasking to provide a more comprehensive measure of screen use. Future studies could also include potential moderating factors, including social economic status or parental rules to further enhance our understanding of relations between screen use, attention and sleep outcomes.

Technology continues to play a growing role in children’s lives and remains a core component of children’s leisure time (Yu and Baxter, 2016). Given that technology has become an integral part of children’s everyday life and can have a positive input on learning, our findings suggest careful management of when a child accesses screen time, supervision of content and amount of time spent on electronic devices could have a beneficial cognitive impact than simply withdrawing access to these devices. Use and availability of modern screen devices have increased, highlighting the critical need for research to remain current with technological trends and their impacts on child development. Our findings indicate that higher levels of screen use do not detrimentally affect behavioural or cognitive attention in children. Instead, tablet and videogame use were associated with better sustained attention performance. Nevertheless, higher tablet use appears to be adversely related to sleep quality. Given that early childhood is a critical period for establishing long-term healthy behaviours, further research is needed to improve understanding of how screen use affects sleep and attention outcomes across development, and its pathways to cognitive and health outcomes.

## Data Availability Statement

The raw data supporting the conclusions of this article will be made available by the authors, without undue reservation.

## Ethics Statement

The studies involving human participants were reviewed and approved by University of Melbourne Human Research Ethics Committee Department of Education and Training Victorian dioceses of the Catholic Education Office. Written informed consent to participate in this study was provided by the participants’ legal guardian/next of kin.

## Author Contributions

KMC and KJ designed and directed the project. RA and KC were involved in the planning and main conceptual ideas of the manuscript. KJ, KMC, and KC wrote the manuscript. KC and FL conducted data collection. KJ conducted all statistical analyses. All authors reviewed the final manuscript.

## Funding

This work was supported by the Australian Research Council [DP170103522] under the Discovery Project Grant awarded to KMC and KJ.

## Conflict of Interest

The authors declare that the research was conducted in the absence of any commercial or financial relationships that could be construed as a potential conflict of interest.

## Publisher’s Note

All claims expressed in this article are solely those of the authors and do not necessarily represent those of their affiliated organizations, or those of the publisher, the editors and the reviewers. Any product that may be evaluated in this article, or claim that may be made by its manufacturer, is not guaranteed or endorsed by the publisher.
